# Gut microbes improve prognosis of *Klebsiella pneumoniae* pulmonary infection through the lung-gut axis

**DOI:** 10.3389/fcimb.2024.1392376

**Published:** 2024-06-05

**Authors:** Yuxiu Tang, Liquan Chen, Jin Yang, Suqing Zhang, Jun Jin, Yao Wei

**Affiliations:** ^1^ Department of Intensive Care Unit, the First Affiliated Hospital of Soochow University, Suzhou, China; ^2^ Department of School of Biology & Basic Medicine Sciences, Suzhou Medical College of Soochow University, Suzhou, China

**Keywords:** *Klebsiella pneumoniae*, FMT, gut microbes, pulmonary infection, drug resistance gene, antibiotheraphy

## Abstract

**Background:**

The gut microbiota plays a vital role in the development of sepsis and in protecting against pneumonia. Previous studies have demonstrated the existence of the gut-lung axis and the interaction between the gut and the lung, which is related to the prognosis of critically ill patients; however, most of these studies focused on chronic lung diseases and influenza virus infections. The purpose of this study was to investigate the effect of faecal microbiota transplantation (FMT) on *Klebsiella pneumoniae*-related pulmonary infection via the gut-lung axis and to compare the effects of FMT with those of traditional antibiotics to identify new therapeutic strategies.

**Methods:**

We divided the mice into six groups: the blank control (PBS), pneumonia-derived sepsis (KP), pneumonia-derived sepsis + antibiotic (KP + PIP), pneumonia-derived sepsis + faecal microbiota transplantation(KP + FMT), antibiotic treatment control (KP+PIP+PBS), and pneumonia-derived sepsis+ antibiotic + faecal microbiota transplantation (KP + PIP + FMT) groups to compare the survival of mice, lung injury, inflammation response, airway barrier function and the intestinal flora, metabolites and drug resistance genes in each group.

**Results:**

Alterations in specific intestinal flora can occur in the gut of patients with pneumonia-derived sepsis caused by *Klebsiella pneumoniae*. Compared with those in the faecal microbiota transplantation group, the antibiotic treatment group had lower levels of proinflammatory factors and higher levels of anti-inflammatory factors but less amelioration of lung pathology and improvement of airway epithelial barrier function. Additionally, the increase in opportunistic pathogens and drug resistance-related genes in the gut of mice was accompanied by decreased production of favourable fatty acids such as acetic acid, propionic acid, butyric acid, decanoic acid, and secondary bile acids such as chenodeoxycholic acid 3-sulfate, isodeoxycholic acid, taurodeoxycholic acid, and 3-dehydrocholic acid; the levels of these metabolites were restored by faecal microbiota transplantation. Faecal microbiota transplantation after antibiotic treatment can gradually ameliorate gut microbiota disorder caused by antibiotic treatment and reduce the number of drug resistance genes induced by antibiotics.

**Conclusion:**

In contrast to direct antibiotic treatment, faecal microbiota transplantation improves the prognosis of mice with pneumonia-derived sepsis caused by *Klebsiella pneumoniae* by improving the structure of the intestinal flora and increasing the level of beneficial metabolites, fatty acids and secondary bile acids, thereby reducing systemic inflammation, repairing the barrier function of alveolar epithelial cells, and alleviating pathological damage to the lungs. The combination of antibiotics with faecal microbiota transplantation significantly alleviates intestinal microbiota disorder, reduces the selection for drug resistance genes caused by antibiotics, and mitigates lung lesions; these effects are superior to those following antibiotic monotherapy.

## Introduction

Sepsis is defined as life-threatening organ dysfunction caused by a dysregulated host response to infection (Sepsis 3.0) (Singer et al., 2016). Approximately 20%-30% of patients die from the disease each year (Fleischmann et al., 2016), and sepsis is regarded as an urgent public health problem by the World Health Organization (WHO) (Rhee et al., 2019). Immune dysregulation often occurs during sepsis as a result of an excessive and uncontrollable inflammatory response, causing secondary nosocomial infections as well as multiorgan dysfunction (Delano and Ward, 2016). The lung is the most vulnerable organ to sepsis-related inflammation. More than 50% of patients have complications such as acute lung injury (ALI) or acute respiratory distress syndrome (ARDS), which are among the leading causes of death in patients with sepsis (Lelubre and Vincent, 2018). Moreover, pneumonia itself is the most common clinical cause of sepsis (Samuelson et al., 2015; Schuijt et al., 2016). *Klebsiella pneumoniae* is one of the most common pathogens involved in septic pneumonia (Nair et al., 2013), and it has been estimated that approximately 3%-8% of all hospital-acquired bacterial infections are caused by *K. pneumoniae* (Ashurst and Dawson, 2023). Due to its high virulence and antibiotic resistance, this bacterium often exhibits a tenacious ability to survive, causing severe systemic symptoms and high morbidity and mortality rates (Liu et al., 2022); furthermore, its global transmission has become a recognized threat.

Antibiotics are still powerful weapons for the treatment of sepsis. Approximately 75% of ICU patients receive antibiotic treatment daily (Vincent et al., 2009). However, with the extensive use of antibiotics in the clinic, antibiotic resistance thereby increasing the difficulty of treating infections; moreover, epidemiological studies have shown that the use of large amounts of antibiotics can also change the composition and diversity of the gut microbes, causing dysfunction of the intestinal microbiology and immune imbalance. Moreover, with damage to the intestinal barrier, opportunistic pathogens and harmful substances that proliferate in the intestine are transferred to the circulatory system causing a systemic inflammatory response (Knoop et al., 2016; Han et al., 2021) and increasing the risk of death; this effect has been verified in septic mice (Panpetch et al., 2018). This adverse effect persists for a long time. Mu et al. (2022) reported that CRKP accounted for 41.2% of secondary infections caused by displaced flora, ranking first in hospital infections. Therefore, we need to find new therapeutic strategies other than antibiotics.

Over the past few decades, the role of the intestinal microenvironment, which is composed of the gut microbes, its metabolites and intestinal mucosal immunity, in maintaining the stability of the organism has been fully recognized, with the gut microbes being the most critical (Van Den Elsen et al., 2017). The gut microbes not only participates in nutrient metabolism in the intestine but also has “colonization resistance”, i.e., targeting pathogens through direct competition for nutrients and the production of substances that inhibit the growth of pathogens to provide intestinal barrier protection; additionally, the gut microbes acts through the activation of the body’s immune response to prevent the invasion of foreign microorganisms. The normal microbiota plays an important role in regulating the balance of various systems in the host, and its disruption is closely related to the occurrence and development of systemic diseases. In addition, the crosstalk between the gut and the lungs has received extensive attention from scholars. As research has progressed, gastrointestinal alterations can also be found in individuals with chronic lung diseases, such as COPD (Bowerman et al., 2020), as well as those with acute lung infections, such as melioidosis (Iapichino et al., 2017), confirming the two-way dialogue between the intestine and the lung.

Previous experiments have proven the protective role of the gut microbes in treating bacterial pneumonia caused by *Streptococcus pneumoniae* (Schuijt et al., 2016), *Pseudomonas aeruginosa* (Wen et al., 2022), and *Escherichia coli* (Chen et al., 2011) and in treating viral pneumonia caused by influenza viruses. By altering the composition of the gut microbes and adjusting the level of intestinal SCFAs, the activity of immune cells can be altered, and the lung immune environment can be remodeled, thereby reducing lung inflammatory responses and injury. However, most studies have focused on the use of antibiotic-treated sterile mouse models or specific probiotics, such as common *Lactobacillus* and *Bifidobacterium* species, as therapeutic means. Although some therapeutic effects can be achieved in animal experiments, the effect of probiotic interventions in clinical patients is not optimistic. Wypych et al. (2019) suggested that this difference may be because different probiotics need to be used in specific environments to function; therefore, the use of community-associated complex microbial agents rather than single strains as a therapeutic method was proposed. The classical approach, namely, fecal microbiota transplantation (FMT), in which normal feces are processed in a certain way and transferred to the patient’s gastrointestinal tract to re-establish the structure of the gut microbes, thereby restoring host function (Porcari et al., 2023), has been demonstrated to restore disturbed intestinal microecosystems and associated functional networks; FMT can prevent the colonization of antibiotic-resistant bacteria in the intestine while adjusting the gut microbes, thereby decreasing the risk of infection (Saïdani et al., 2019). Although for the successful treatment of sepsis has been reported, investigations into the treatment of this disease are still in the experimental stage, and the mechanisms have not been fully verified. Therefore, based on the principle of the gut-pulmonary axis, combined with the current clinical need for new antibiotic treatment due to the emergence of resistance in *K. pneumoniae*, we designed this study to explore the differences in the therapeutic effects of antibiotics and FMT on the inflammatory response and local pathological changes in *K. pneumoniae*-induced sepsis in mice. To this end, we combined a mouse model and multi-histological analysis to clarify the role of the lung-gut axis with sepsis and to determine its effect on prognosis to provide new ideas for the treatment of *K. pneumoniae* in the future.

## Results

### Changes in the gut microbes induced by pulmonary infection with *Klebsiella pneumoniae* and associated antibiotic and fecal microbiota transplantation therapy

**Figure 1:**
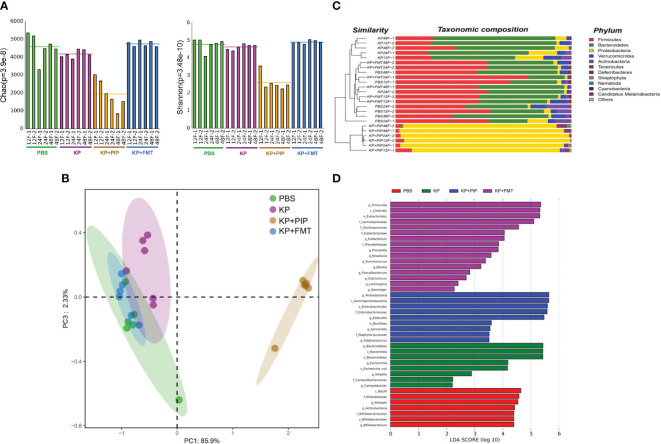
**(A)**. Fecal microbiota transplantation appropriately restores changes in gut microbes diversity induced by pulmonary infections. Fresh feces from mice of PBS, KP, KP+PIP and KP+FMT group at 12, 24 and 48 hours were obtained for metagenomic sequencing and the diversity of the gut microbes in each group was compared using the Mothur calculation (n = 6). **(B)**. The composition of gut microbes was significantly different after antibiotic treatment. Principal component analysis (PcoA) was performed using R vegan, thus visualizing the differences in gut microbes β-diversity between PBS, KP, KP+PIP and KP+FMT group (n = 6). **(C)**. The gut microbes is dominated by opportunistic pathogens after antibiotic treatment at the phylum level. Based on the Bray-Curtis algorithm, QIIME was used to calculate the distance between the samples to obtain the Bray-Curtis distance matrix, based on which hierarchical clustering analysis was performed, and then unweighted group averaging algorithm was used to construct a tree structure for visual analysis (n = 6). **(D)**. The intestinal flora is dominated by favorable flora after fecal microbiota transplantation, whereas opportunistic pathogens predominate after antibiotic therapy. Differences in the composition of gut microbes from PBS, KP, KP+PIP and KP+FMT group at the genus level were analyzed using LEfSe (LDA Effect Size) analysis to look for differential flora between the groups (n = 6).

Increasing evidence shows that there is a close relationship between the gut microbes and various systemic diseases (Tanaka et al., 2021; Spence, 2023), and identification of the gut-lung axis highlights the correlation between the gut microbes and lung diseases (Budden et al., 2017). We used metagenomic sequencing analysis to observe the changes in gut microbes in each group. The results showed that the diversity of the gut microbes was lower in the pneumonia-derived sepsis group than in the normal group. The most significant decrease in the diversity of the gut microbes was observed after treatment with antibiotics, while the changes induced by lung infection could be appropriately restored after FMT (**Figure 1A**). β-Diversity analysis (**Figure 1B**), as reflected in the PCA, revealed that the structure of the gut flora in the antibiotic group differed significantly from that in the other three groups, the gut flora in the KP+FMT group was similar to that in the control group, and the diversity of the gut flora in the KP group was also different from that in the KP+FMT group and the PBS group. Bar graph species composition analysis was performed to investigate the differences in the relative abundance of gut microbes in each group of mice. At the phylum level (**Figure 1C**), the KP+PIP group had a significantly increased abundance of *Proteobacteria* and *Tenericutes*, and the abundances of *Firmicutes* and *Bacteroidetes* were significantly lower than those in the KP+FMT group; moreover, the intestines of the KP+FMT group were dominated by *Firmicutes* and *Bacteroidetes*, which was similar to the main composition of the intestinal bacteria in the PBS group. LEfSe and the Bray−Curtis distance were used to analyze the different bacteria between the groups, and linear discriminant analysis (LDA) was used to estimate the bacteria with significantly different abundances between groups. A comparison at the genus level revealed (**Figure 1D**) that the abundances of *Campylobacter*, *Escherichia coli* and *Shigella* in the KP group were greater than those in the PBS group. There were significantly more opportunistic pathogens, such as *Klebsiella*, *Staphylococcus* and *Salmonella*, in the probiotic group than in the KP group after antibiotic treatment. However, beneficial gut microbes, such as *Lachnospira*, *Ruminococcus*, *Eubacterium* and *Coprococcus*, were dominant after FMT. The above results showed that antibiotic treatment can significantly promoted the disruption of the composition of the intestinal microbiota induced by pulmonary infection, thereby causing a substantial increase in opportunistic pathogens in the intestine; however, FMT stabilized the structure of the gut microbes and appropriately restored the alteration to the gut microbes caused by *K. pneumoniae* pulmonary infection.

### Fecal microbiota transplantation confers protection against pneumonia-induced sepsis caused by *K. pneumoniae* in mice

**Figure 2:**
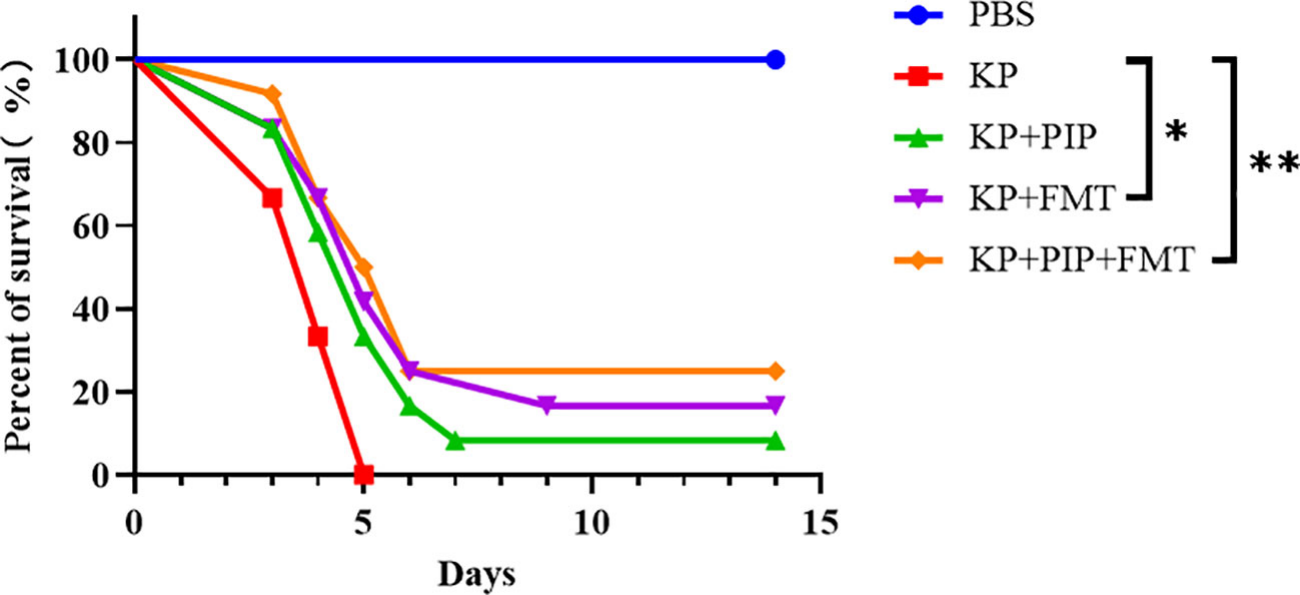
Both fecal microbiota transplantation and post antibiotic fecal microbiota transplantation were effective in improving mortality rates. Prism software was used to create survival curves by Kaplan and Meier’s product limit method and to compare the survival curves of PBS, KP, KP+PIP and KP+FMT groups using the log-rank test and Gehan - Wilcoxon test(* p<0.05, ** p<0.01)(n = 6).

To compare the therapeutic efficacy of antibiotics and FMT for pneumonia-induced sepsis caused by *K. pneumoniae*, we used intranasal inoculation of *K. pneumoniae* to cause pulmonary infection and induce sepsis. Antibiotics, FMT agents, or a combination of the two were administered 24 hours after pulmonary infection to observe the difference in survival rate between the groups. Compared with those in the KP group, the 14-day morbidity was lower in both the antibiotic and FMT groups (**Figure 2**), and there was a significant difference in the survival rate of the KP+FMT group compared with that of the KP+PIP+FMT group and the KP group (P<0.05). The survival rate of the KP+FMT group was slightly higher than that of the KP+PIP group, but the difference was not significant; however, the combination of the two treatments effectively increased the survival rate and improved the clinical prognosis of mice.

### Fecal microbiota transplantation reduces lung inflammatory damage associated with pneumonia caused by *K. pneumoniae*

**Figure 3:**
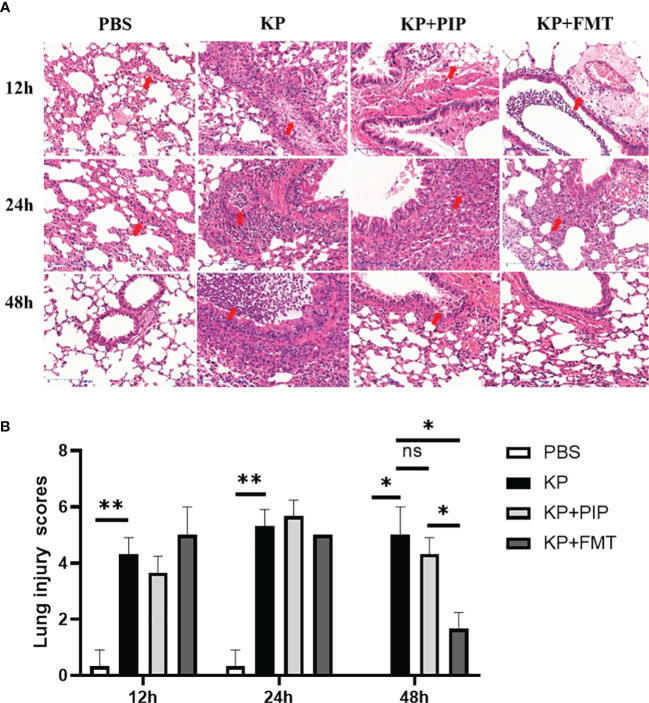
**(A)**. Fecal microbiota transplantation and antibiotics are effective in ameliorating local pathological damage in the lungs. HE staining was used to stain lung tissue sections of PBS, KP, KP+PIP and KP+FMT groups to observe the local pathological damage of lungs in each group (n = 6), magnification: ×200, scale bar = 100 μm. **(B)**. Faecal microbiota transplantation improves pulmonary local pathological injury more than antibiotic therapy. The Smith semi-quantitative scoring system was used to quantify five aspects of lung oedema, alveolar and interstitial hemorrhage, alveolar and interstitial inflammation, lung atelectasis and hyaline membrane formation in the HE staining results of the lung tissues of each group of mice (n = 6)(ns, P>0.05, * P<0.05, ** P<0.01).

We next evaluated the protective effects of antibiotics and FMT against pneumonia caused by *K. pneumoniae*. As shown in **Figure 3A**, thickening of the alveolar walls, widening of the alveolar septum, partial alveolar atrophy with massive neutrophil infiltration, loosely arranged connective tissues with lymphocyte and neutrophil infiltration, exfoliated epithelial cells and eosinophilic secretion, as well as massive infiltration of inflammatory cells, were observed in the bronchioles of the KP group. In the antibiotic group, many eosinophilic secretions in the lungs were observed at 12 h and 24 h. The connective tissue around the blood vessels was loose, the structure was disrupted, and the alveolar wall was thickened. The alveolar septum was widened, and alveolar atrophy was common. With time, the inflammatory changes observed via lung pathology at 48 hours improved. A few inflammatory cells and secretions were observed in the bronchus, and the alveolar wall was slightly thickened. In the KP+FMT group, inflammatory cells in the bronchioles, perivascular oedema, and thickening of the alveolar wall were also observed at 12 h and 24 h, but the pathological structure tended to normalize at 48 hours. The bronchioles were structurally intact, and the alveolar wall consisted of a monolayer of epithelium with a clear structure. No obvious pathological changes were observed in the group. Pathology analysis based on the Smith-semiquantitative lung injury score yielded a correlation pathology score graph (**Figure 3B**), which showed that with the prolongation of time, both antibiotics and FMT appropriately reduced pathological lung damage at 48 h and FMT more significantly ameliorated inflammatory damage in the lungs than antibiotics (P < 0.05).

### Fecal microbiota transplantation reduces the systemic inflammatory response associated with pneumonia caused by *K. pneumoniae* and restores lung airway barrier function

**Figure 4:**
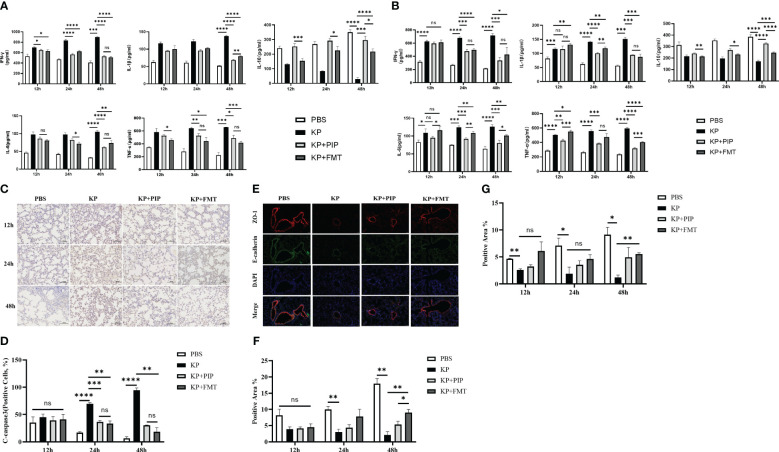
**(A)**. The ability of antibiotic treatment to reduce pro-inflammatory factors and increase anti-inflammatory factors in serum is greater than that of fecal microbiota transplantation. The levels of pro-inflammatory factors TNF-ɑ, IFN-γ, IL-6 and IL-1β and anti-inflammatory factor IL-10 in serum of mice at 12, 24 and 48 hours between PBS, KP, KP+PIP and KP+FMT groups were compared (n = 6)(ns, P>0.05, * P<0.05, ** P<0.01, *** P<0.001, **** P<0.0001). **(B)**. The ability of antibiotic treatment to reduce pro-inflammatory factors and increase anti-inflammatory factors in alveolar lavage fluid is greater than that of fecal microbiota transplantation. The levels of pro-inflammatory factors TNF-ɑ, IFN-γ, IL-6 and IL-1β and anti-inflammatory factor IL-10 in alveolar lavage fluid of mice at 12, 24 and 48 hours between PBS, KP, KP+PIP and KP+FMT groups were compared (n = 6)(ns, P>0.05, * P<0.05, ** P<0.01, *** P<0.001, **** P<0.0001). **(C)**. Both fecal microbiota transplantation and antibiotic therapy reduce the expression of apoptotic protein cleaved-caspase 3 in the airway mucosal barrier. The expression of the apoptotic protein cleaved-caspase 3 in alveolar epithelial cells of mice in PBS, KP, KP+PIP and KP+FMT groups were determined by immunohistochemistry at 12,24 and 48 hours (n = 6).Yellow color indicates positive cells and blue color indicates negative cells, magnification: ×200, scale bar = 100 μm. **(D)**. Fecal microbiota transplantation is more effective than antibiotic therapy in reducing the expression of apoptotic protein cleaved-caspase 3 in alveolar epithelial cells. Positive cell counts were performed under a light microscope in three fields of vision in 12-, 24 and 48 hour immunohistochemical sections in PBS, KP, KP+PIP and KP+FMT groups. Positive cell count ratios were used to represent the expression level of the apoptotic protein Cleaved-Caspase3 in each group (n = 6) (ns, P>0.05, ** P<0.01, *** P<0.001, **** P<0.0001). **(E)**. Both fecal microbiota transplantation and antibiotic therapy elevate connexin expression in the airway mucosal barrier. Immunofluorescence analysis of the airway mucosal barrier protein expression of ZO-1 and E-cadherin in PBS, KP, KP+PIP and KP+FMT groups at 48 hours (n = 6). Red labels represent ZO-1, green labels represent E-cadherin, magnification: ×200, scale bar = 100 μm. **(F)**. Fecal microbiota transplantation is more effective than antibiotic treatment in elevating the levels of expression of ZO-1 in the airway mucosal barrier. Images were collected under a fluorescence microscope, and three fields of vision were selected at 12, 24, and 48 hours for PBS, KP, KP+PIP and KP+FMT groups. Positive cell areas were calculated, and positive area ratios were used to represent the levels of expression of ZO-1 in each group (n = 6) (ns, P>0.05, * P<0.05, ** P<0.01). **(G)**. Fecal microbiota transplantation effectively restores the levels of expression of airway mucosal barrier connexin E-cadherin induced by lung infection. Images were collected under a fluorescence microscope, and three fields of vision were selected at 12, 24, and 48 hours for PBS, KP, KP+PIP and KP+FMT groups. Positive cell areas were calculated, and positive area ratios were used to represent the levels of expression of E-cadherin in each group (n = 6) (ns, P>0.05, * P<0.05, ** P<0.01).

We compared the effects of two treatments, antibiotics and FMT, on the levels of inflammatory factors, including the proinflammatory factors TNF-ɑ, IFN-γ, IL-1b, and IL-6, and the anti-inflammatory factor IL-10, in the serum and alveolar lavage fluid of pneumonia-induced sepsis model mice (**Figures 4A, B**). The results showed that, compared with those in the KP group, both antibiotics and FMT were effective at reducing the levels of proinflammatory factors and increasing the levels of anti-inflammatory factors. Overall, antibiotics led to a slightly greater decrease in proinflammatory factor levels such as IL-1β in the serum and TNF-ɑ in the alveolar lavage fluid, and increase in anti-inflammatory factor levels than did FMT (P < 0.05). The expression of the apoptotic protein Cleaved-Caspase3 in alveolar epithelial cells was measured via immunohistochemistry (**Figure 4C**), and the expression levels of intercellular junction proteins, including ZO-1 in tight junctions and E-cadherin in adherent junctions, in the airway mucosal barrier were determined via immunofluorescence (**Figure 4E**). The changes in the function of the airway mucosal barrier were quantified (**Figures 4D, F, G**). As time progressed, the expression level of apoptotic proteins in alveolar epithelial cells in the KP group was significantly greater than that in the normal group, while the expression level of connexin in the airway mucosa was significantly lower. Both antibiotic and FMT treatments restored pulmonary infection-induced epithelial cell apoptosis and increased connexin protein expression, which mainly manifested as an increase in the level of the tight junction protein ZO-1, and the effect of FMT was slightly greater than that of antibiotic treatment (P < 0.05).

### Antibiotic therapy induces gut microbes disorders, thereby decreasing fatty acid and secondary bile acid levels, whereas fecal microbiota transplantation appropriately restores the metabolite alterations caused by pulmonary infection

**Figure 5:**
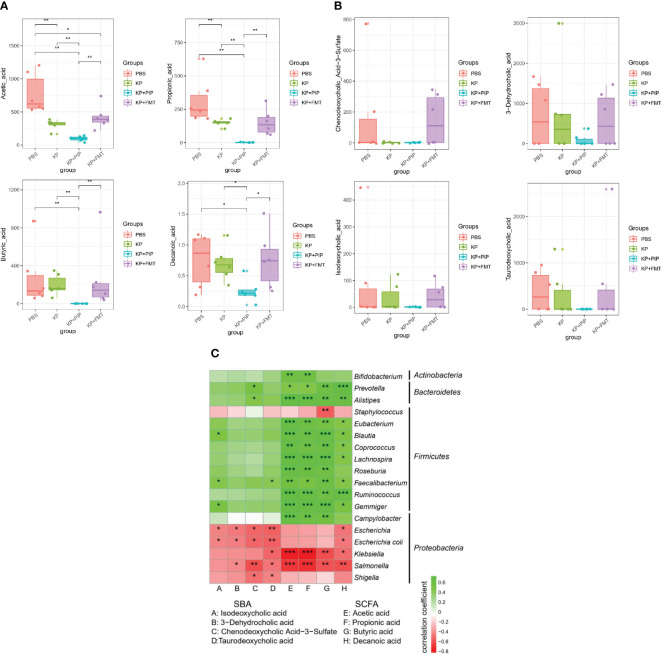
**(A)**. Fecal microbiota transplantation is effective in restoring levels of beneficial intestinal metabolites of fatty acids induced by lung infection relative to antibiotic therapy. Levels of beneficial intestinal metabolites fatty acids were determined by macrogenomics in PBS, KP, KP+PIP and KP+FMT groups (n = 6). Significant differences were found in acetic acid, propionic acid, butyric acid and propionic acid (* P<0.05, ** P<0.01). **(B)**. Fecal microbiota transplantation is effective in restoring levels of beneficial intestinal metabolites of secondary bile acids induced by lung infection relative to antibiotic therapy. Levels of beneficial intestinal metabolites secondary bile acids were determined by macrogenomics in PBS, KP, KP+PIP and KP+FMT groups (n = 6). Significant differences were found in chenodeoxycholic acid 3-sulfate, isodeoxycholic acid, taurodeoxycholic acid and 3-dehydrocholic acid. **(C)**. Fecal microbiota transplantation can increase the level of beneficial metabolites in the gut by improving the structure of the gut microbes, thereby increasing the level of beneficial metabolites in the gut. Heatmap was used to represent the correlation between specific differential gut microbes and metabolites between PBS, KP, KP+PIP and KP+FMT groups (n = 6). The horizontal axis represents differential metabolites, the vertical axis represents differential flora, green is a positive correlation, red is a negative correlation, and the darker the color, the greater the correlation (* P<0.05, ** P<0.01, *** P<0.001).

The intestinal tract is the largest digestive organ, and intestinal bacteria produce hundreds of microbial metabolites by decomposing nutrients such as dietary fiber, fat, protein and other nutrients, thereby regulating host metabolism and maintaining the balance of the host (Dang and Marsland, 2019; Funabashi et al., 2020; Guo et al., 2023); among these metabolites, fatty acids account for a large proportion of the metabolites (Koh et al., 2016; Dang and Marsland, 2019). A comparison of the fatty acid composition between the groups (**Figure 5A**) showed that in the KP group, the levels of the main short-chain fatty acids, acetic acid and propionic acid, decreased significantly, and that of butyric acid also decreased, but these decreases were relatively insignificant compared with those of the other two short-chain fatty acids. Comprehensive analysis of the four groups revealed that the levels of the three main short-chain fatty acids in the KP+PIP group were significantly lower than those in the other three groups, while those in the KP+FMT group were significantly greater than those in the KP+PIP group; thus, FMT could appropriately reverse the reduction in intestinal short-chain fatty acid production caused by pulmonary infection. At present, little is known about the role of medium-chain fatty acids. In this study, a comparison of caprylic acid, which is the most important regulator, revealed a trend similar to that of short-chain fatty acids. In addition to fatty acids, another common metabolite in the intestine is bile acid. The liver metabolizes cholesterol to produce primary bile acid, which combines with taurine and glycine to enter the gallbladder. When the body is stimulated by feeding, bile acid enters the intestine with bile, and 95% of the metabolite enters the entero-hepatic circulation, while the remaining 5% will be transformed into secondary bile acid by modifying the gut microbes (Cai et al., 2022); this process inhibits the overgrowth of bacteria in the intestine, reduces the damage to the ileal mucosa, and enhances the antibacterial defense of the small intestine (Long et al., 2017; Sinha et al., 2020; Bamba et al., 2022). By comparison (**Figure 5B**), the levels of the intestinal secondary bile acids chenodeoxycholic acid 3-sulfate, isodeoxycholic acid, taurodeoxycholic acid and 3-dehydrocholic acid after pneumonia caused by *K. pneumoniae* were lower than those in the PBS group and decreased compared to those in the normal control group; in particular, the different isomer formation of chenodeoxycholic acid 3-sulfate and 3-dehydrocholic acid significantly decreased, while it significantly increased after FMT. After antibiotic treatment, as the gut microbes changed, the intestinal secondary bile acid concentration decreased significantly, while there was a trend towards recovery of the secondary bile acid concentration in the KP+FMT group compared to that in the KP group. Therefore, the results showed that antibiotics may cause a substantial decrease in the production of favorable metabolites in the intestine due to a significant reduction in the number of beneficial bacteria, whereas FMT may have a therapeutic effect on pulmonary infections by restoring changes in the gut microbes and increasing the production of fatty acids and secondary bile acids. The correlations of specific differentially abundant flora and metabolites in each group were analyzed (**Figure 5C**), and a negative correlation (p<0.05) was found between the flora that was significantly more abundant in the KP group and the KP+PIP group; for example, *Klebsiella*, *Salmonella*, and *Escherichia* were associated with the differentially abundant metabolites found above. The main differentially abundant flora in the KP+FMT group, such as *Ruminococcus* and *Eubacterium*, were positively correlated with the metabolites (p<0.05). The above results indicate that antibiotic treatment can reduce the production of beneficial metabolites by changing the structure of the gut microbes; for example, by reducing the content of beneficial flora and increasing the content of adverse flora. However, FMT can restore the structure of the gut microbes caused by pulmonary infection and increase the level of beneficial metabolites, thus improving patient prognosis.

### Fecal microbiota transplantation restores gut flora disorder caused by antibiotic treatment and reduces the abundance of drug resistance genes

**Figure 6:**
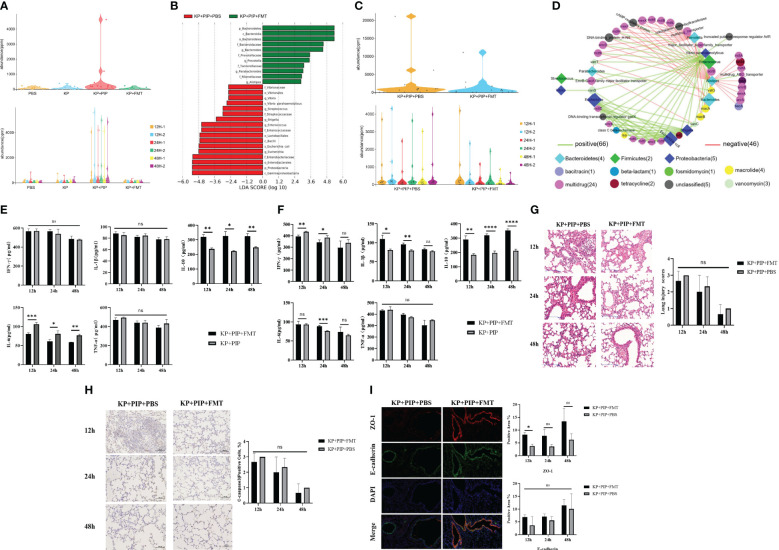
**(A)**. Antibiotic treatment significantly increases levels of drug-resistant genes in the gut of mice. Violinplot was used to represent the abundance of drug-resistance genes in the total gut microbiota of each group in PBS, KP, KP + PIP and KP + FMT groups (n = 6), and the comparison of the abundance of drug-resistance genes in the gut microbiota of the individual samples in each group. **(B)**. Combined fecal microbiota transplantation after antibiotics is effective in improving gut microbiological structure. Differences in the composition of gut microbes from KP+PIP+PBS and KP+PIP+FMT groups were analyzed using LEfSe (LDA Effect Size) analysis to look for differential flora between the groups (n = 6). **(C)**. Combined fecal microbiota transplantation after antibiotics is effective in reducing the abundance of drug-resistance genes generated during antibiotic therapy. Violinplot was used to represent the abundance of drug-resistance genes in the total gut microbiota of KP+PIP+PBS and KP+PIP+FMT groups (n = 6), and the comparison of the abundance of drug-resistance genes in the gut microbiota of the individual samples in each group. **(D)**. After treatment with antibiotics combined with fecal microbiota transplantation, the differential gut microbes of mice was negatively correlated with drug-resistance genes. Correlation analysis of differential gut microbes and drug-resistance genes between groups KP+PIP+PBS and KP+PIP+FMT groups using bubble connectivity plots, with positive correlation in green and negative correlation in red (n = 6). **(E)**. Combined fecal microbiota transplantation after antibiotics had a greater ability to reduce pro-inflammatory factors as well as elevate anti-inflammatory factors in serum than antibiotic monotherapy. The levels of pro-inflammatory factors TNF-ɑ, IFN-γ, IL-6 and IL-1β and anti-inflammatory factor IL-10 in serum of mice at 12, 24 and 48 hours between KP+PIP+PBS and KP+PIP+FMT groups were compared (n = 6)(ns, P>0.05, * P<0.05, ** P<0.01, *** P<0.001). **(F)**. Combined fecal microbiota transplantation after antibiotics had a greater ability to reduce pro-inflammatory factors as well as elevate anti-inflammatory factors in alveolar lavage fluid than antibiotic monotherapy. The levels of pro-inflammatory factors TNF-ɑ, IFN-γ, IL-6 and IL-1β and anti-inflammatory factor IL-10 in alveolar lavage fluid of mice at 12, 24 and 48 hours between KP+PIP+PBS and KP+PIP+FMT groups were compared (n = 6)(ns, P>0.05, * P<0.05, ** P<0.01, *** P<0.001, **** P<0.0001). **(G)**. Combined fecal microbiota transplantation after antibiotics reduces pulmonary local pathological damage similarly to antibiotic monotherapy. HE staining was used to stain lung tissue sections of KP+PIP+PBS and KP+PIP+FMT groups to observe the local pathological damage of lungs in each group (n = 6), magnification: ×200, scale bar = 100 μm. The Smith semi-quantitative scoring system was used to quantify five aspects of lung oedema, alveolar and interstitial hemorrhage, alveolar and interstitial inflammation, lung atelectasis and hyaline membrane formation in the HE staining results of the lung tissues of each group of mice (n = 6)(ns, P>0.05, * P<0.05, ** P<0.01). **(H)**. Combined fecal microbiota transplantation after antibiotics reduces apoptotic proteins cleaved-caspase 3 in airway epithelial cells similarly to antibiotic monotherapy. The expression of the apoptotic protein cleaved-caspase 3 in alveolar epithelial cells of mice in KP+PIP+PBS and KP+PIP+FMT groups were determined by immunohistochemistry (n = 6), magnification: ×200, scale bar = 100 μm. Positive cell count ratios were used to represent the expression level of the apoptotic protein Cleaved-Caspase3 in each group (n = 6) (ns, P>0.05). **(I)**. Combined fecal microbiota transplantation after antibiotics elevate connexin expression in the airway mucosal barrier similarly to antibiotic monotherapy. Immunofluorescence analysis of the airway mucosal barrier protein expression of ZO-1 and E-cadherin in KP+PIP+PBS and KP+PIP+FMT groups at 48 hours, magnification: ×200, scale bar = 100 μm. Positive cell areas were calculated, and positive area ratios were used to represent the levels of expression of ZO-1 **(A)** and E-cadherin **(B)** in each group (n = 6) (ns, P>0.05, * P<0.05).

A comparison of antibiotics and FMT revealed that antibiotic treatment led to a greater reduction of the systemic inflammatory response in patients with sepsis caused by pulmonary infection than FMT, but the treatment process caused intestinal microbiome disturbance and reduced the production of beneficial metabolites, fatty acids and secondary bile acids. A comparison of the levels of drug resistance genes in the intestine revealed that antibiotics damaged the structure of the intestinal microbiota and caused a significant increase in the expression of drug resistance genes in the intestine (**Figure 6A**). However, FMT treat lung infection by restoring the intestinal microecological balance and therefore is unlikely to cause an increase in drug resistance genes. Therefore, in the present study, we readministered FMT at the time when the changes in gut microbes and drug resistance genes were most obvious after antibiotic treatment in pneumonia-derived sepsis caused by *K. pneumoniae* to explore whether the combination of the two treatments could improve pulmonary infection and reduce the expression of drug resistance genes produced during antibiotic treatment. The results showed that the increase in opportunistic pathogens such as *Enterococcus*, *Staphylococcus*, *Shigella*, and *Escherichia* was still predominant in the KP+PIP+PBS group, whereas significant restoration of the gut microbes structure was observed after antibiotic treatment with FMT, as demonstrated by the increase in favorable gut microbes such as *Bacteroides*, *Alistipes*, and *Prevotella*; there was also a reduction in the level of resistance genes induced during antibiotic treatment in comparison to those in the KP+PIP+PBS group (**Figures 6B, C**). The correlation between the differentially abundant flora and the major resistance genes between the two groups was analyzed (**Figure 6D**). There was a significant correlation between the drug resistance genes and the specific flora present in the KP+PIP+PBS group, whereas the abundance of favorable flora after FMT was negatively correlated with drug resistance genes. Moreover, by comparing the two groups in terms of the overall therapeutic effect on mice with pneumonia-derived sepsis cause d by *K. pneumoniae* (**Figures 6E–I**), the combination of the two treatments had greater effects on the levels of host inflammatory factors, pulmonary inflammatory injury, and mucosal barrier integrity in the lung airways than antibiotic monotherapy; however, the difference between the two groups was not significant. Therefore, the combination of antibiotics and FMT has an additive effect in the treatment of sepsis induced by pulmonary infection.

## Discussion

Pneumonia remains the leading cause of death and hospitalization worldwide, especially among children and elderly individuals (Bengoechea and Sa Pessoa, 2019). *K. pneumoniae* is one of the most common pathogens isolated, and antibiotic treatment is still the main clinical treatment. As mentioned above, antibiotic treatment can control bacterial infection, but it may also drive the development of drug resistance in non-susceptible bacteria, alter the composition of the intestinal microbiota and its diversity, create conditions for the upregulated expression and rapid spread of antibiotic resistance genes, and may also damage the intestinal barrier integrity, resulting in bacterial translocation and dysregulation of the immune response. The proposal of the gut-pulmonary axis opens new avenues for clinical treatment. The gut microbes can strengthen the resistance and clearance of pathogens in the lungs by regulating the signaling pathways involved in the body’s immune response and reducing or delaying the occurrence and development of respiratory diseases. The intestinal microecology is closely related to respiratory diseases. However, the efficacy of using gut microbes to treat pneumonia caused by *K. pneumoniae*, as well as its difference from traditional antibiotic treatment, are still unclear. Therefore, we used a sepsis model of pulmonary infection caused by intranasal inoculation of *K. pneumoniae* to explore the effect of the gut-pulmonary axis on pulmonary infection and compared the differences in therapeutic efficacy among antibiotics, FMT, and antibiotics and FMT combination in pulmonary infection.

The results showed that, compared with those in the control group, the proinflammatory factor levels in the serum and alveolar lavage fluid were significantly greater in the pneumonia group, while the anti-inflammatory factor IL-10 was significantly lower. There were obvious inflammatory changes in the lungs and a certain change in the gut microbes, with a slight decrease in the abundance and diversity of the flora compared with those in the normal control group; these changes mainly manifested as a decrease in *Firmicutes* abundance and an increase in *Proteobacteria* abundance. At the genus level, over time, an increase in opportunistic pathogens such as *Campylobacter*, *Escherichia coli* and *Shigella* was observed in the intestines of the KP group, with the gradual emergence of intestinal manifestations due to pulmonary infection. *Firmicutes* is the main bacterial species that colonize and ferment dietary fiber in the large intestine. It has been proved to be the main producer of short-chain fatty acids, an important metabolite in the intestine, and contributes to intestinal health (Sun et al., 2023). In contrast, the significantly increased gut microbes in the KP group, such as *Campylobacter*, is the leading cause of foodborne gastroenteritis worldwide, which can invade the underlying tissue of intestinal epithelial cells, such as the lamina propria, into the bloodstream and reach different organs, such as the spleen, liver or mesenteric lymph nodes. Colonization in sterile mice can cause damage to the immune response in the body (Backert et al., 2013; O'Loughlin et al., 2015). Liang (Liang et al., 2022) and Sun (Sun et al., 2020) et al. found that the levels of opportunistic pathogens in the intestine of CLP-induced septic mice were significantly increased, such as *Klebsiella* and *Shigella*, which disrupted the intestinal microecological balance and promoted the growth of inflammatory levels in the body, which was similar to the results of this experiment.

In contrast to the findings of other studies, most previous studies exploring the role of the gut microbes in pulmonary infections used antibiotics in combination with pre-experimental intestinal preparation of mice to remove the gut microbes and observe the tolerance of mice to sepsis infection, thus demonstrating the importance of the gut microbes (Dessein et al., 2020; Wen et al., 2022); however, the experiments in this study were performed using antibiotics to treat *K. pneumoniae* infection, which is more in line with the normal procedure used for clinical treatment. We found that after antibiotic treatment, the level of inflammation in the mice decreased significantly, and the level of IL-10 increased compared to that in the KP group; however, the diversity of the gut microbes was also significantly lower than that in the KP group. In the intestine, the abundance of Proteobacteria, mainly *K. pneumoniae* and *Enterobacter*, which are the most common opportunistic pathogens in the intestine, increased significantly. However, under normal conditions, due to the colonization resistance of the gut microbes, which can activate the immune response of the organism and thus target the removal of pathogens through competition for living space and nutrients (Tängdén and Giske, 2015; Wypych et al., 2019), proliferation is inhibited. When the body undergoes inflammatory pathological damage or antibiotic treatment, the intestinal microbiota is damaged, the competitive exclusion of pathogens is weakened, and many opportunistic pathogens emerge as the dominant flora of the intestine (Van Nood et al., 2013; Kim et al., 2017; Shimasaki et al., 2019; Lou et al., 2022). Compared with antibiotic treatment, the administration of FMT after pulmonary infection reduced inflammation levels in mice compared to those in the KP group, but the reductions in the levels of serum and lung proinflammatory factor, such as IL-1β, and increases in the anti-inflammatory factor IL-10 were slightly less than those following antibiotic treatment. However, the amelioration of pathological damage to lung inflammation tended to be similar to that seen after antibiotic treatment, and FMT restored the intestinal diversity and alterations caused by lung infection to a level similar to that of the normal gut microbes.

The difference in the effect on inflammation may be because antibiotics, as an effective treatment for infection, can enter the circulatory system after absorption and thus act on the whole body; thus, the reduction in inflammatory factor levels is greater following antibiotic treatment than following FMT. This effect occurs through the restoration of the gut microbes by acting on the intestinal tract and the use of the gut-pulmonary axis to alleviate lung lesions. The pathway of action of FMT is more complicated, and the onset time may be relatively prolonged and have a lag effect; therefore, the observation time can be extended into the next steps of the experiment to compare the long-term therapeutic effects of the two treatments after pulmonary infection. However, the therapeutic effect of antibiotics on lung lesions and the effects on the intestinal microbiota are similar to the conclusions proposed by previous researchers. In animal studies, antibiotics lead to the consumption of certain gut microbes, which increases the risk of airway disease and viral pulmonary infection. After intragastric administration of antibiotics to mice (Sherwin et al., 2017; Di Cicco et al., 2018), Lou et al. (2022) reported that the abundance of *Bacteroidetes* and *Firmicutes* decreased significantly, while the abundance of *Proteobacteria* increased; they also reported an increase in *Shigella*, while antibiotics changed the body’s metabolic pathways, such as the pathways of tyrosine, aspartic acid and arachidonic acid metabolism. However, after FMT, the abundance of *Proteobacteria* was significantly reduced, and the microbial abundance and diversity were increased in antibiotic-treated mice; thus, the composition of the body’s intestinal microbiota was restored.

The most important pathways through which antibiotics disrupt the gut microbes and thus increase the risk of sepsis development, as well as death, are mediated by intestinal metabolites and by a reduction in short-chain fatty acids (SCFAs), which are the source of nutrients for intestinal cells; the reduction in SCFA levels leads to a loss of intestinal mucosal barrier function and a reduction in the ability to defend against foreign invasions (Yamada et al., 2015; Baggs et al., 2018). Wu et al. (2020) reported that in mice infected with *K. pneumoniae*, the levels of *Bifidobacteria* and *Clostridium*, which produce SCFAs in the intestine, were significantly reduced, which aggravated pathological lung damage. By supplementing with SCFAs, the bacterial load in the lungs can be reduced, lung infection can be improved, and the survival rate can increase. Vieira et al. (2016) summarized the characteristics of host changes after pulmonary infection with *K. pneumoniae* and reported that surviving cells in the lungs quickly produced the chemokine CXCL1, which led to the accumulation of neutrophils in the lung, activation of the cytokine cascade reaction and the production of reactive oxygen species, thereby exacerbating lung infection. Clarke (2014) demonstrated that the recovery of the gut microbes after pulmonary infection with *K. pneumoniae* can enhance the phagocytic function of alveolar macrophages and reactive oxygen species-mediated bactericidal capacity via the Nod receptor and SCFAs, which are key factors in the interaction between the gut microbes and host. This leads to the expression of lower amounts of CXCL1 by macrophages in the lung, thereby reducing the inflow of neutrophils and reducing lung injury (Trompette et al., 2018). In this study, we also observed that the acetic acid, propionic acid and butyric acid levels, which are common intestinal SCFAs in *K. pneumoniae*-treated mice, were lower than those in the PBS group. In addition, we also found several specific intestinal metabolites, such as decanoic acid, which is a medium-chain fatty acid; and chenodeoxycholic acid 3-sulfate, isodeoxycholic acid, taurodeoxycholic acid; and 3-dehydrocholic acid, which are secondary bile acids. These levels were lower in the intestines of mice infected with *K. pneumoniae* than in those of mice infected with PBS, and these levels substantially decreased after antibiotic treatment but were restored after FMT. Interestingly, in our study, after FMT, the butyric acid concentration was slightly lower than that in the KP group but still significantly greater than that in the KP+PIP group. We speculate that this difference occurred because after pulmonary infection in the KP group, the lung-intestinal axis acted on the gut microbes for a relatively long period of time, and the final time of sampling in our experiment was 48 hours. At this time, the impact of pulmonary infection on the gut microbes might not have reached the peak point in time, so the disruption of the gut microbes was less common; this result also be seen in the gut microbes structure of the KP group. Therefore, the change in butyric acid production is small, and if the intestinal mucous membrane barrier is damaged at this time, then the use of butyric acid as the energy source for epithelial cells in the colon will be relatively reduced; thus, butyric acid in the intestine will accumulate relatively well, and the residual butyric acid content will increase (Morrison and Preston, 2016; Liu et al., 2018). After FMT, the gut microbes of mice with pulmonary infection were restored to levels similar to that of the normal intestinal microflora, and the amount of butyric acid produced increased. However, in differentiated colonocytes, butyric acid is rapidly oxidized and used for cellular energy production, as well as for promoting the expression of connexin in the intestinal mucosal barrier, thereby restoring intestinal mucosal barrier function (Liu et al., 2018; Gai et al., 2021; Lou et al., 2022). Therefore, the KP+FMT group exhibited a decrease in butyrate levels compared with the KP group. Zhao et al. (2021) reported that supplementation of mouse intestinal medium-chain fatty acids (MCFAs) with decanoic acid could upregulate the expression of G protein-coupled receptor 43 (GPR-43) in the intestine, thereby enhancing intestinal antioxidant capacity and intestinal barrier function, adjusting the structure of the intestinal microbiota, and restoring the health of the host intestine. The study also demonstrated that the administration of decanoic acid could decrease the inflammatory response, improve host immune function, increase the production of SCFAs in the intestine, regulate the intestinal microenvironment, and inhibit the proliferation of harmful microbes, thus increasing host defense (Xu et al., 2018; Zicker et al., 2019; Zhao et al., 2021). Similar changes were also found in the present study, where a decrease in the level of decanoic acid in the intestine was observed in the KP group, and the production of decanoic acid obviously increased with the improvement in the gut microbes after FMT, in contrast to that after antibiotic treatment.

Another important metabolite in gut microbes-host metabolism is bile acid, of which isodeoxycholic acid (iDCA), the 3β-OH differential isomer of deoxycholate (DCA), was found by Thanissery et al. (2017) to inhibit spore germination, alter growth, and inhibit the toxin activity of *Clostridium difficile* strains; the level of DCA was significantly reduced in high-fat diet-fed obese rats (Lin et al., 2019). Taurodeoxycholic acid is formed by combining deoxycholic acid and taurine. Chen et al. (2019) reported that taurodeoxycholic acid can reduce the activation of relevant proteins in the apoptotic pathway and thus inhibit apoptosis, and taurodeoxycholic acid can inhibit the expression of endoplasmic reticulum stress (ERS)-related proteins, thus reducing the level of IL-17 produced by Th17 cells in the lung and serum during human herpes simplex virus type 1 (HSV1) infection, attenuating lung inflammation damage and improving prognosis. Therefore, combined with the findings of previous studies, the therapeutic effect of FMT on *K. pneumoniae* may be achieved by improving the structure of the gut microbes and increasing the production of favorable fatty acid and secondary bile acid metabolites; this leads to dampening of the systemic inflammatory response, a decrease in pathological inflammatory damage in the lung, and improvement of the function of the pulmonary airway barrier to treat pulmonary infection caused by *K. pneumoniae* and improve patient prognosis. However, during antibiotic treatment, many opportunistic pathogens in the intestine proliferate and significantly reduce the level of favorable metabolites.

Antibiotic resistance (AMR) occurs when microorganisms, such as bacteria and viruses, can fight the drugs that kill them. This is a naturally occurring process (Bhopale, 2020). The emergence of AMR prevents microorganisms from responding to antibiotics, greatly reducing the availability and effectiveness of antibiotics and increasing the severity of disease and the risk of death (Kalra et al., 2022). An RCT showed that patients who died of sepsis had significantly more antibiotic-resistant bacterial species, such as *Clostridium* and *Enterococcus*, than did those who survived (Shimizu et al., 2018). Long-term observation of drug resistance in patients has indicated that spontaneous decolonization can occur in only 9% of patients without intervention, and drug resistance will persist in non-decolonized patients (O'Fallon et al., 2009). According to the World Health Organization, if left unchecked, the number of deaths caused by AMR may increase to 10 million by 2050 and AMR will become one of the major public health threats (Samreen et al., 2021; Unemo et al., 2021). The gut serves as a host for drug-resistant bacteria to infect organisms, and the intestinal microbiota is a repository of antibiotic resistance genes (ARGs) (Campbell et al., 2020); therefore, decolonization of antibiotic-resistant bacteria by acting on the intestinal microbiota has become a feasible approach. Studies have shown that FMT, while restoring the diversity of the microbiota, also plays a role in reducing the drug resistance of the gut microbes and promoting the decolonization of drug-resistant bacteria. For example, FMT can significantly decrease the number and downregulate the expression of drug resistance genes in methicillin-resistant *Staphylococcus aureus* (MRSA), vancomycin-resistant *Enterococcus* (VRE) and broad-spectrum b-lactamase (ESBL)-producing bacteria (Saïdani et al., 2019). In half of patients treated, FMT can decolonize antibiotic-resistant bacteria in the intestine of colonized adults with a relatively low risk (Tavoukjian, 2019). In this study, based on the analysis of drug resistance genes in each group, we found that the number of drug resistance genes in the intestine increased significantly after antibiotic administration, and the proportion of multidrug resistance genes was the greatest, while the number of intestinal drug resistance genes in the group treated with FMT alone was similar to that in the normal control group. After antibiotic treatment, the expression of drug resistance genes in the intestine significantly decreased, and the improvements in inflammation and pathological lung damage were more significant, indicating that FMT was effective. Moreover, the administration of flora transplantation after antibiotic treatment significantly reduced the expression of drug resistance genes in the intestine, suggesting that FMT achieved decolonization of organisms with antibiotic-resistance genes.

In the 14-day mortality observation of five groups of mice in this study, it was found that the antibiotic treatment group, the fecal microbiota transplantation treatment group and the combination of the two groups could reduce the mortality rate of the mice compared with the KP group, among which the mortality rate of the KP+FMT group and the KP+PIP+FMT group differed significantly from that of the KP group (P<0.05), but the difference was relatively small. Compared with the pulmonary local pathological damage, the function of the airway mucosal barrier, the gut microbes and the level of metabolites in each group, it was found that the fecal microbiota transplantation treatment showed a significant improvement in the early 48 hours, which was different from the long-term 14-day morbidity and mortality rate results. For this difference, it may be due to the pathogenesis of sepsis infection itself is an unbalanced immune response, and the early body is in the acute inflammatory response period after the occurrence of sepsis induced by pulmonary infection, which is related to the occurrence of early death (van der Poll et al., 2017; van der Poll et al., 2021). While the early pro-inflammatory response to the invading pathogens or danger signals, it will induce the body ‘s immunosuppressive response, which involves a variety of cell types and factors. Dickson (Dickson et al., 2016) et al. have found that CD8+ T-cells in patients with sepsis have a weakened proliferative capacity, enhanced apoptosis, and a weakened cytotoxicity, and the effect does not change with the conversion of early pro-inflammatory response to the anti-inflammatory response, leaving the body in a state of continuous immunocompromised dysfunction. Ost (Ost et al., 2016) et al. found the emergence of myeloid-derived suppressor cells (MDSCS) can hinder immune function through a variety of mechanisms, including deprivation of L-arginine (necessary for T cell function), stimulation of TERG-cell expansion, and suppression of macrophage and dendritic-cell function. The existence of this immunosuppressive state is closely related to the increased susceptibility of patients with sepsis to secondary infection, so that the latent pathogens, mainly the virus, are activated and infect the body (Ong et al., 2017). At this time, the body enters the chronic reaction period of sepsis, which is mainly manifested as Persistent Inflammation, Immunosuppression, and Catabolism Syndrome (PICS), which is related to the delayed death of the organism (Darden et al., 2021; Chadda and Puthucheary, 2024). This may explain the obvious effect of early treatment, but the difference in long-term mortality is relatively small. At the same time, considering that this is mainly an animal experiment, and in clinical practice, our treatment of sepsis caused by pulmonary infection is not only limited to monotherapy with antibiotics or fecal microbiota transplantation, but also combined with sputum drainage and other organ function support. By improving the relevant clinical experiments, we may observe a significant improvement in the mortality of clinical patients. In addition, the number of mice in each group was small in the observation experiment of mortality (n = 12). Considering that the strain is a highly virulent Klebsiella pneumoniae, its own mortality rate is high. Therefore, the mortality rate of 14 days after fecal microbiota transplantation is less different from that of antibiotic treatment group. We will explore it in the next experiment.

## Conclusions

In the present study, FMT treatment increased the production of beneficial metabolites in the intestines while improving the intestinal microbial balance in mice such as fatty acids and secondary bile acids, thereby reducing the body’s inflammatory response and attenuating localized pathological damage to the lungs, restoring lung airway barrier function and improving prognosis. There is a correlation between the balance of flora and inflammatory injury in the body, which further supports that adjusting the gut microbes has therapeutic significance for pulmonary infection. Compared with antibiotic monotherapy, it was found that OMC in combination with antibiotics can significantly downregulate the expression of drug resistance genes produced during antibiotic treatment. Therefore, in our study, we showed that FMT can be used as a therapeutic measure for pneumonia-derived sepsis caused by *Klebsiella pneumoniae* or as an adjuvant after antibiotic treatment. Using the beneficial flora and metabolites is a viable strategy as there were no obvious differences found in the experiment. Although drugs play a certain role in the treatment of pulmonary infection, additional animal and clinical studies are needed to prove the feasibility and safety of combining antibiotics with FMT.

## Limitations

The following limitations of this study should be noted. 1. Long-term observation should be carried out in further experiments to clarify the continuous changes in pulmonary-induced sepsis caused by *K. pneumoniae*. 2. In this study, antibiotics used in similar experiments were selected according to previous methods and found to be relatively homogeneous, and other antibiotic regimens may have different effects. There may be differences in the flora of mice from different suppliers, and the reactivity to antibiotics, as well as FMT, may also be inconsistent. 3. There may be some differences in microbial composition and clearance rate between adult mice and elderly mice. Further research should consider more closely matching the clinical situation as pneumonia occurs more often in elderly people.

## Methods

### Mice

All mouse experiments were conducted in accordance with the guidelines of the Animal Care and Utilization Committee of Soochow University Institutions. C57BL/6 mice were purchased from Soochow University Laboratory Animal Center, Jiangsu Province, and were housed in Soochow University’s P2 Animal Room under standard care at 23–25°C with free access to food and water. All mice were males, 6–8 weeks old, and acclimatized for 1 week prior to the start of the experiments.

### Mouse models of *K. pneumoniae* infection

In the present study, *K. pneumoniae* (ATCC 43816) was used to induce pneumonia-induced sepsis in mice via intranasal inhalation according to previous methods (Claushuis et al., 2018; Perlee et al., 2019; Qin et al., 2022). *K. pneumoniae* was purchased from the American Type Culture Collection (ATCC) official website of the United States and cultured in the etiology laboratory of Soochow University. The solution was stored at -80°C and removed for resuscitation, culture inoculation, and the determination of the bacterial concentration when needed. During the experiment, the mice were anaesthetized with 2% sodium pentobarbital (50 mg/kg), 1×10^4^ CFU of *K. pneumoniae* was mixed with 50 µL of phosphate-buffered saline (PBS), and after the bacteria were instilled through the nose, the mice were placed vertically for 30 seconds to enhance delivery to the lungs. The control group received 50 µl of PBS, and all mice were euthanized at the end of the experiment.

### Animal experiment

The mice were randomly divided into six groups of six mice each. The mice were divided into the following groups: the PBS group; the blank control group; the KP group, the pneumonia-derived sepsis group; the KP+PIP group; and the pneumonia-derived sepsis + antibiotic group. At 24 hours after nasal inhalation of *Klebsiella pneumoniae*, piperacillin (300 mg/kg) was injected subcutaneously, and the one-day dose was divided into two injections separated by at least 12 hours for 3 days (Trautmann et al., 1986; Rice et al., 2004). In the KP+FMT group and the pneumonia-derived sepsis + FMT group, 24 hours after nasal inhalation of *Klebsiella pneumoniae*, 0.3 ml of fecal microbiota was administered by gavage once a day for 3 days (**Supplementary Figure S1**). In the KP+PIP+PBS group, three consecutive days of antibiotic treatment were administered, followed by three consecutive days of gavage with PBS solution at one-day intervals. In the KP + PIP + FMT group and the pneumonia-derived sepsis+ antibiotic + FMT group, first, a pre-experiment was carried out to observe the microbial structure and the production of drug resistance genes in the intestines of the KP+PIP group. The time at which the most obvious change was observed was selected as the time point for FMT following antibiotic administration; that is, on the second day of the three consecutive days of antibiotic administration (see **Supplementary Figures S3**-**S5** for the results of the pre-experiment), *Klebsiella pneumoniae* was injected subcutaneously with piperacillin (300 mg/kg) 24 hours after nasal inhalation modelling, with one day’s dose divided into two injections separated by an interval of at least 12 hours for three days. After an interval of one day, the FMT solution was given once a day at a rate of 0.3 ml for three days (**Supplementary Figure S2**). The mice in each group were sacrificed at 12, 24 and 48 hours after the experiment to collect relevant samples.

### Fecal microbiota transplantation solution

Fresh feces were collected from healthy C57BL/6 adult mice (6–8 weeks old), and 5 g of fresh feces was homogenized in 50 mL of sterile PBS (1:10 ratio) and centrifuged at 2000 rpm for 5 minutes to collect the fecal supernatant. Glycerol was added to a final concentration of 10%, after which the solution was stored at -80°C and removed when needed. The KP+FMT and KP+PIP+FMT groups were intragastrically administered 0.3 ml of the various substances at the indicated time points once a day for three consecutive days (Liu et al., 2020; Lou et al., 2022).

### Stool, lung tissue and alveolar lavage fluid

At each time point, fresh feces were taken from the mice; the samples were subsequently placed in sterilized 1.5 ml EP tubes, quickly frozen in liquid nitrogen, and stored at -80°C. In accordance with previous experimental methods (Qin et al., 2022), after blood was taken from the eyeball, the mice were sacrificed by cervical dislocation, and the mice were subsequently fixed on the experimental operating table and opened via the abdomen. The thoracic cavity of the mice was opened, and the lungs were fully exposed and separated. After, the left bronchus and blood vessels were ligated by using surgical sutures, the left lungs were removed and preserved in 4% paraformaldehyde, and the right lung was freed. The skin on the neck was cut and bluntly separated to fully expose the trachea. A disposable mouse tracheal tube was inserted into the trachea, and a 1 ml syringe was passed through the tracheal tube. Afterwards, the tissue was injected with 0.5 ml of sterile PBS, and the solution was slowly extracted from the injected PBS in sterile EP tube on ice for preservation; this process was repeated twice, after which the right lungs of the mice were gently squeezed and the tissue was fully gavaged. The sampling was completed, and the samples were stored at -80°C until use.

### HE staining and pathologic analysis

The left lungs of the mice were removed and placed in 4% paraformaldehyde for adequate fixation. The tissues were subsequently dehydrated, embedded, and sectioned in paraffin, and 4 µm sections were obtained. After dewaxing and hydration, HE staining was performed with hematoxylin and eosin, after which the sections were dehydrated and sealed under a microscope. Pathological analysis was performed according to Smith’s semiquantitative lung injury pathological scoring criteria as detailed in previous studies (Smith et al., 1997; Wang et al., 2023).

### Cytokine measurement

The whole blood of mice was centrifuged to obtain serum, combined with alveolar lavage fluid obtained by lung lavage of mice. The concentration of TNF-α, IL-6, IL-10, IL-1β and IFN-γ in the samples was determined by double antibody sandwich ELISA according to the instructions on the 96-well enzyme-linked immunosorbent assay kit (J & L Biological, China). The serum and alveolar lavage fluid were diluted 5-fold, and added to the ELISA plate in order. After incubation, the prepared 1: 100 biotinylated antibody working solution and enzyme conjugate working solution were added in turn. After incubation and washing, the chromogenic substrate was added to the plate until it appeared blue, and then the termination solution was added to become yellow. The OD value was measured at the wavelength of 450 nm by microplate reader. The concentration of inflammatory factors in the corresponding samples was calculated by the standard curve drawn by the standard substance. Finally, the level of inflammatory factors in each group was obtained by multiplying the dilution multiple (5-fold).

### Immunohistochemistry

The 4 µm paraffin-embedded lung tissue sections were dewaxed and hydrated, and antigen retrieval was carried out via the microwave heating repair method. After the samples were washed with PBS, endogenous catalase activity was blocked, the serum was sealed, the primary antibody was added after the sealing solution was removed and the samples were incubated overnight. Then, the secondary antibody corresponding to the primary antibody was added dropwise and the samples were incubated at room temperature. Finally, freshly prepared DAB color development solution was added dropwise, and the duration of color development was controlled under a microscope. After rinsing and restaining with hematoxylin, the slides were dehydrated, cleared and sealed for microscopic examination, after which the number of positive cells was counted.

### Immunofluorescence

After dewaxing and hydration, the paraffin sections were placed in EDTA antigen retrieval solution (pH 9.0) for antigen retrieval via the microwave heating method. The sections were subsequently washed in PBS and incubated with 3% BSA for 30 min. Afterwards, the corresponding primary antibody was added, and the samples were incubated overnight at 4°C. After washing with PBS solution, the slides were incubated with secondary antibody for 50 minutes in the dark and then subjected to nuclear staining with DAPI, after which the autofluorescence quencher was added for 5 minutes. After rinsing with running water, the slices were blocked, and images were collected for analysis.

### Metagenomic sequencing

With the Illumina HiSeq sequencing platform, a small fragment library was constructed for paired-end sequencing. Fecal samples stored at -80°C were subjected to DNA extraction using the Fast DNA Spin Kit for Feces (MPBIO, CA, USA) according to the manufacturer’s instructions. The qualified DNA samples were randomly broken into small fragments of approximately 300 bp using a Covaris M220 ultrasonic crusher, after which library preparation was completed. The constructed library was initially quantified by Qubit and diluted to 2 ng/µl. Then, an Agilent 2100 was used to analyze the inserted fragments in the library. After the insertion fragments met expectations, the effective concentration of the library was accurately determined via qPCR. Finally, different libraries were pooled according to the effective concentration and target downstream data volume and sequenced on the sequencing platform. After obtaining the non-redundant gene set through the metagenomic sequencing, the gene sequence was annotated with the drug resistance gene database SARG to obtain the corresponding drug resistance gene type. Then, combined with the gene abundance table, the abundance of drug resistance genes in each sample was obtained. Finally, using statistical test methods, the types of drug resistance genes between groups can be obtained.

### Statistical analysis

Statistical analyses were performed using QIIME1, R language software and GraphPad Prism 8.0.1. The mean ± standard deviation was used to assess quantitative data, and t tests or two-way analysis of variance (ANOVA) and Bonferroni *post hoc* correction were used to analyze the data instead of individual comparisons. The Chao1 and Shannon indices were utilized to assess the α diversity using Mothur. Then, based on the Bray−Curtis algorithm, QIIME was used to calculate the distance between each sample to obtain the Bray−Curtis distance matrix. According to the distance matrix, hierarchical clustering analysis was performed, and the unweighted group average algorithm was subsequently used to construct a tree structure for visual analysis. Based on the calculated inter sample distances, principal component analysis (PCOA) was performed using the R package vegan to visualize the differences in intestinal microecological β diversity among the samples. The probability relationships between the grouping categories and the actual distributions of the samples were inferred from the sample distributions to analyze the overall differences between the two groups of samples at the species level. LEfSe was used to analyze the species that differed between the groups, and the Spearman rank correlation coefficient was calculated for correlation analysis. All the results between groups were analyzed using a statistical significance level of P < 0.05.

## Data availability statement

The datasets presented in this study can be found in online repositories. The names of the repository/repositories and accession number(s) can be found in the article/**Supplementary Material**.

## Ethics statement

The animal study was approved by the Animal Care and Utilization Committee of Soochow University Institutions. The study was conducted in accordance with the local legislation and institutional requirements.

## Author contributions

YT: Conceptualization, Data curation, Formal analysis, Investigation, Methodology, Visualization, Writing – original draft, Writing – review & editing. LC: Conceptualization, Data curation, Formal analysis, Investigation, Methodology, Writing – original draft. JY: Conceptualization, Formal analysis, Investigation, Methodology, Writing – review & editing. SZ: Data curation, Investigation, Methodology, Resources, Software, Writing – review & editing. JJ: Conceptualization, Formal analysis, Methodology, Project administration, Supervision, Validation, Visualization, Writing – review & editing. YW: Conceptualization, Formal analysis, Funding acquisition, Project administration, Supervision, Validation, Visualization, Writing – review & editing.
