# Supplementary material for: Gut microbes improve prognosis of Klebsiella pneumoniae pulmonary infection through the lung-gut axis
Source: Front Cell Infect Microbiol. 2024 Jun 5;14:1392376. doi: 10.3389/fcimb.2024.1392376 (PMC11188585; doi:10.3389/fcimb.2024.1392376)

Supplementary Information

Gut microbes improve prognosis of *Klebsiella pneumoniae* pulmonary infection through the lung-gut axis

Yuxiu Tang[1, a], Liquan Chen[1, a], Jin Yang[a], Suqing Zhang[b], Jun Jin[*,a], Yao Wei[*,a]

[a] **Department of Intensive Care Unit, the First Affiliated Hospital of Soochow University, Suzhou 215100, Jiangsu, China**
[b] **Department of School of Biology & Basic Medicine Sciences, Suzhou Medical College of Soochow University, Suzhou 215123, Jiangsu, China**

[1] **These authors equally contributed to this work.**

[*]**Address correspondence to Yao Wei, dr_betty@126.com; Jun Jin, jinjundoctor@163.com**

Supplementary Fig. S1. Experimental flow chart 1

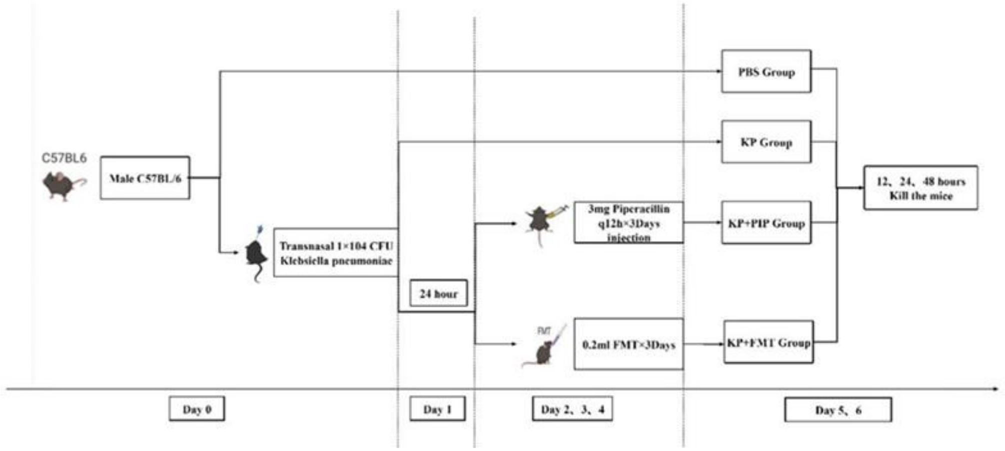

Supplementary Fig. S2. Experimental flowchart 1

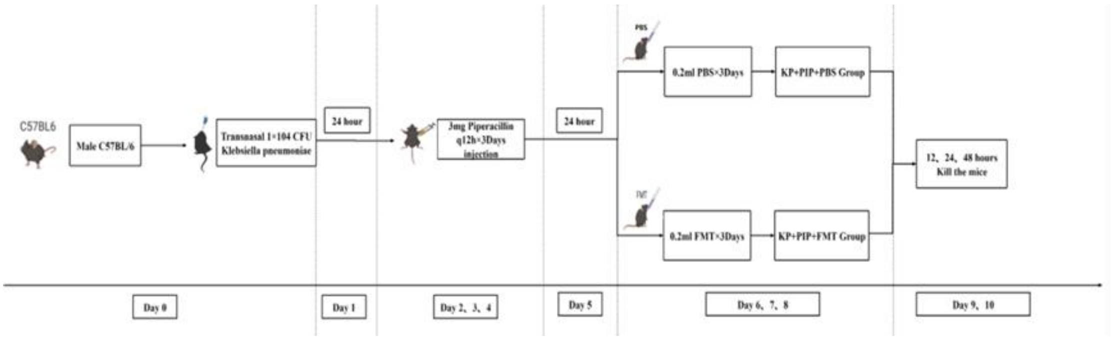

Supplementary Fig. S3. Chao diversity index of the pre-experiment

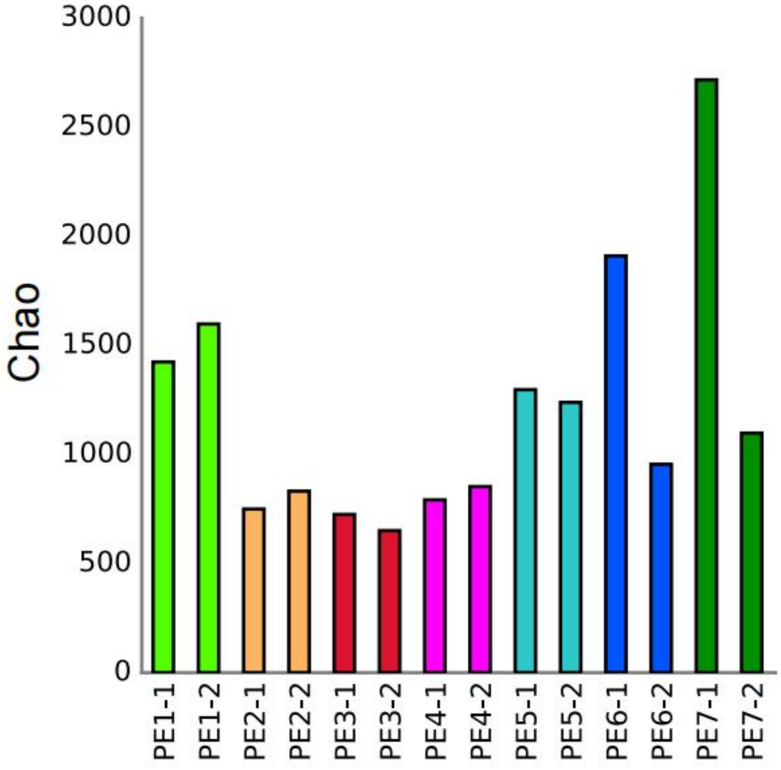

Supplementary Fig. S4. Shannon diversity index of the pre-experiment

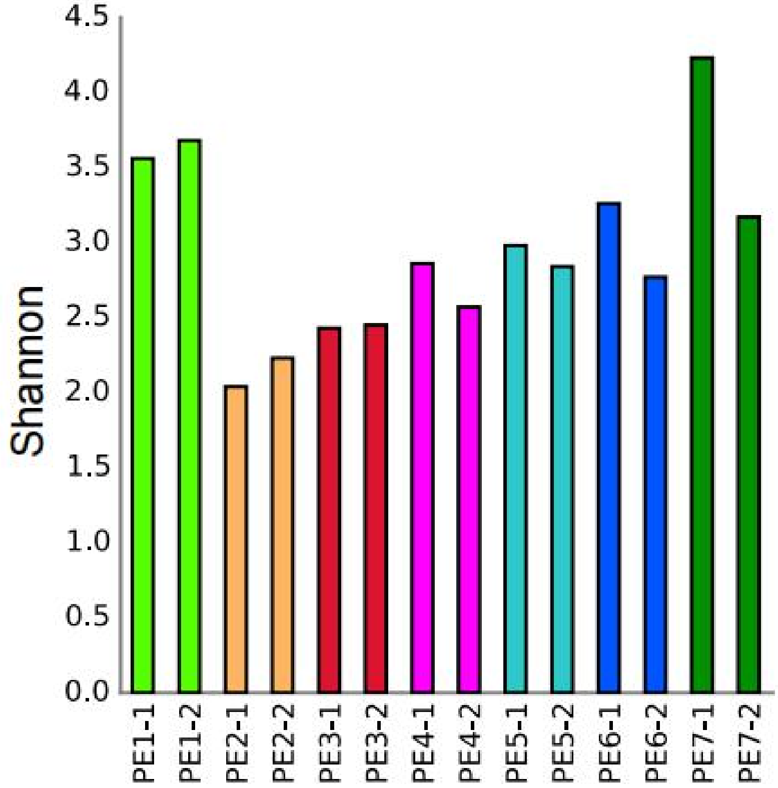

Supplementary Fig. S5. Trend of drug resistance genes in pre-experiment

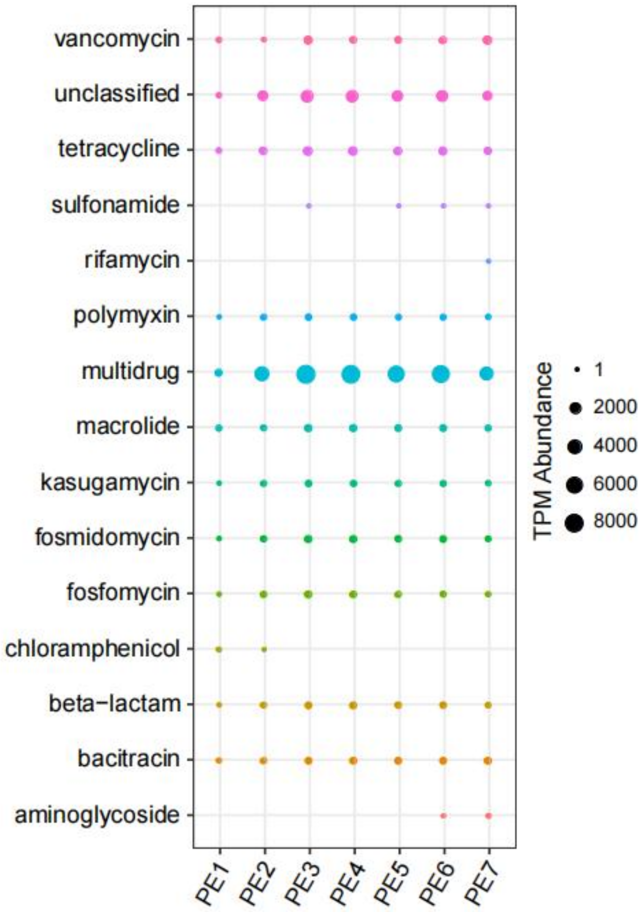

Supplement: Supplementary file 1 [file DataSheet_1.pdf]
